# Assessment of potential biomarkers of subclinical vitamin K deficiency in patients with end-stage kidney disease

**DOI:** 10.1186/2054-3581-1-13

**Published:** 2014-06-24

**Authors:** Meghan J Elliott, Sarah L Booth, Wilma M Hopman, Rachel M Holden

**Affiliations:** Department of Medicine, University of Calgary, Calgary, AB Canada; Jean Mayer USDA Human Nutrition Research Center of Aging at Tufts University, Boston, MA USA; Clinical Research Centre, Kingston General Hospital, Kingston, ON Canada; Division of Nephrology, Department of Medicine, Queen’s University, Kingston, ON Canada; Division of Nephrology, Foothills Medical Centre, 1403 29th Street NW, T2N 2T9 Calgary, AB Canada

**Keywords:** Chronic kidney disease, Hemodialysis, Phylloquinone, PIVKA, Osteocalcin, Vitamin K

## Abstract

**Background:**

A significant proportion of hemodialysis patients have functional, but modifiable, vitamin K deficiency.

**Objective:**

To determine the correlates of poor vitamin K status in hemodialysis patients.

**Design:**

Cross-sectional study.

**Setting:**

Hemodialysis units at Kingston General Hospital and its satellite centres, Ontario, Canada.

**Patients:**

Patients undergoing outpatient hemodialysis for end-stage kidney disease.

**Measurements:**

Serum concentrations of phylloquinone, undercarboxylated prothrombin, also known as protein induced by vitamin K absence or antagonism – factor II (PIVKA-II), and the percentage of undercarboxylated osteocalcin (%ucOC).

**Methods:**

Vitamin K status was determined in fasting blood samples of hemodialysis patients. Bivariate relationships were examined using parametric and non-parametric statistics as appropriate. Multivariable linear regression models were applied to identify predictors of vitamin K status.

**Results:**

Among 44 HD patients, criteria for subclinical vitamin K deficiency were met in 13.6% (phylloquinone < 0.4 nmol/L), 51% (%ucOC > 20%) and 90.9% (PIVKA-II > 2.0 nmol/L) of subjects. Phylloquinone levels were positively associated with total cholesterol, triglyceride levels and non-smoking status. Higher %ucOC was associated with increased calcium-phosphate product. Increased PIVKA-II levels were observed with advancing age, reduced dialysis adequacy, lower HDL and a history of coronary artery disease. There were no associations found among the individual biomarkers of vitamin K status. In a multi-variable model, triglycerides were the only significant predictor of phylloquinone levels, while increasing phosphate and decreasing PTH were independent predictors of %ucOC. PIVKA-II levels increased by 0.54 nmol/L for every 10-year increase in age.

**Limitations:**

Observational study; small sample size.

**Conclusions:**

A significant proportion of HD patients met criteria for subclinical vitamin K deficiency. Of the biomarkers measured, PIVKA-II may be superior given its independence of renal function or dyslipidemia, both of which may confound the other vitamin K biomarkers. Studies in patients with ESKD linking biomarkers of vitamin K status to important patient outcomes, including cardiovascular disease, nutritional status and mortality, are required in order to determine the optimal biomarker for evaluating vitamin K status in this particular population.

## Abrégé

**Contexte:**

Une proportion considérable de patients traités en hémodialyse ont une carence fonctionnelle, bien que modifiable, en vitamine K. L’objectif de cette étude est de déterminer la corrélation entre une carence en vitamine K chez les patients en hémodialyse.

**Méthodes:**

Dans le cadre de cette étude d’observation, le statut de la vitamine K était déterminé dans les échantillons sanguins pris à jeun chez 44 patients en hémodialyse, en mesurant les concentrations de phylloquinone, de prothrombine non décarboxylée, aussi connue comme la protéine induite en l’absence de vitamine K ou par antagonisme du facteur II (PIVKA-II), et le pourcentage d’ostéocalcine non décarboxylée (%ucOC). Les relations bivariées ont été examinées à l’aide de statistiques paramétriques et non paramétriques. Des modèles multivariés à régression linéaire ont été appliqués pour déterminer les prédicteurs du statut de la vitamine K.

**Résultats:**

Les critères de carence infraclinique en vitamine K étaient remplis chez 13,6% (phylloquinone < 0,4 nmol/l), 51% (%ucOC > 20%) et 90,9% (PIVKA-II > 2,0 nmol/l) des sujets. Les taux de phylloquinone ont été positivement associés avec les taux de cholestérol total, et de triglycérides, ainsi que le statut de non-fumeur. Un taux élevé de %ucOC a été associé avec une augmentation du produit phosphocalcique. Des taux croissants de PIVKA-II ont été observés avec l’âge, le caractère non adéquat de la dialyse, un faible taux de HDL et des antécédents de coronaropathie. Aucune association n’a été établie parmi les biomarqueurs individuels du statut de la vitamine K. Dans un modèle multivariable, les triglycérides constituaient le seul prédicteur important des taux de phylloquinone, alors qu’un taux croissant de phosphate et un taux décroissant de PTH étaient des prédicteurs indépendants de %ucOC. Les taux de PIVKA-II ont augmenté de 0,54 nmol/l par tranche d’âge de 10 ans.

**Conclusions:**

Une proportion considérable de patients en hémodialyse ont rempli les critères de carence infraclinique en vitamine K. Parmi les biomarqueurs mesurés, PIVKA-II serait le plus fiable, considérant son indépendance par rapport à la fonction rénale ou la dyslipidémie, lesquels peuvent confondre les autres biomarqueurs de la vitamine K. Des études auprès de patients souffrant d’insuffisance rénale terminale permettraient de créer des liens entre les biomarqueurs du statut de la vitamine K et des résultats importants pour le patient, tels que les maladies cardiovasculaires, l’état nutritionnel et le décès, et seraient donc nécessaires afin de déterminer le biomarqueur optimal pour évaluer le statut de la vitamine K dans cette population en particulier.

## What we know

A significant proportion of hemodialysis patients have functional, but modifiable, vitamin K deficiency.

## What this study adds

Many dialysis patients have sub-clinical vitamin K deficiency and multiple biomarkers can be used to measure vitamin K status in end-stage kidney disease patients. Protein induced by vitamin K absence or antagonism - factor II (PIVKA-II) as a biomarker of vitamin K status has the advantage of being independent of kidney function and lipid profile.

## Background

Cardiovascular disease is the leading cause of death in patients with end-stage kidney disease (ESKD), accounting for approximately 50% of the annual mortality rate of this population [1]. In addition to traditional cardiovascular risk factors such as hypertension, diabetes mellitus and cigarette smoking, kidney-specific risk factors also contribute to this increased cardiovascular risk in ESKD patients. This relates, in part, to abnormal divalent ion mineral metabolism that occurs in the renal population [2]. In particular, these patients demonstrate extensive cardiovascular calcification, including that of coronary arteries and cardiac valves, that progresses more rapidly as their kidney disease deteriorates and has been associated with an increased cardiovascular mortality [3–7]. Matrix Gla protein (MGP) and osteocalcin (OC) are two proteins that regulate mineralization of vascular tissues and bone, respectively [8, 9]. In addition to its critical role in coagulation, vitamin K is a cofactor for post-translational modification that is critical for these proteins to exert their protective biological effects, with evidence of increased mortality among ESKD patients with low levels of carboxylated MGP [10]. The function of these vitamin K dependent proteins (VKDP) is sensitive to vitamin K status, and therefore vitamin K could represent a modifiable target in the management of cardiovascular disease in this patient population.

Phylloquinone (vitamin K1) is the primary North American dietary source and circulating form of vitamin K [11]. In the presence of severe dietary restriction of phylloquinone, there is an increase in the undercarboxylated forms of the VKDPs [12, 13]. Osteocalcin (OC) is a noncollagenous bone matrix protein synthesized by mature osteoblasts. The proportion of OC that is uncarboxylated (%ucOC) has been used extensively in the non-renal population as a sensitive marker of vitamin K status in bone. Subclinical vitamin K deficiency is defined by an increase in %ucOC above 20% and predisposes to reduced bone mineral density and fractures [14–18]. Protein induced by the vitamin K absence or antagonism factor-II (PIVKA-II) is a measure of prothrombin undercarboxylation, with elevated levels corresponding to poorer vitamin K status. In the ESKD population, PIVKA-II measurements have the advantage of not being affected by renal function [19] but until recently had only been measured in dialysis patients with hepatitis C.

Westenfeld *et al* recently evaluated the impact of 3 doses of vitamin K2 on levels of ucMGP, PIVKA-II, %ucOC and vitamin K2 in 53 prevalent hemodialysis patients [20]. They demonstrated a dose-dependent decrease in levels of ucMGP and PIVKA-II, while levels of %ucOC were only changed at the highest dose of vitamin K2. While %ucOC is a useful measure of functional vitamin K status in the general population, the spectrum of mineral and bone disorders and potential for accumulation of OC in those with renal failure may render it a less useful measure in dialysis patients.

These studies indicate that many hemodialysis patients have functional, but modifiable, vitamin K deficiency. Examining the correlates of poor vitamin K status in dialysis patients might therefore aid in identifying those individuals at risk. At present, no single biomarker is a robust measure of vitamin K sufficiency and deficiency. Therefore, multiple biomarkers are used where possible, each of which reflects a different aspect of vitamin K intake, absorption, transport or function as a co-factor for the gamma-carboxylation of VKDPs. Therefore, the purpose of this study was determine the correlates of three estimates of vitamin K status (phylloquinone levels, PIVKA-II and %ucOC) in a prevalent cohort of Canadian hemodialysis patients and to assess the association between these circulating measures of vitamin K status in individuals with end-stage kidney disease.

## Methods

### Study population

This cross-sectional study included 44 patients receiving hemodialysis (HD) for irreversible chronic kidney disease (CKD) at Kingston General Hospital, Ontario, Canada or one of its satellite dialysis centers. Consecutive patients greater than 18 years of age were approached during one of their scheduled outpatient hemodialysis sessions. All patients provided informed consent according the Declaration of Helsinki. Procedures were in accordance with the ethical standards of Queen’s University and approved by the Tufts Medical Center Institutional Review Board (Boston, MA). Of 123 patients considered for this study, 44 consented for enrollment and none subsequently withdrew. Subjects were excluded from the study protocol if they were unwilling (n = 21) or unable (n = 10) to provide consent; if they had been exposed to warfarin (a vitamin K antagonist) within the preceding year (n = 44); or if they were hospitalized at the time of enrollment (n = 4).

### Analytical measures

Clinical data were abstracted by patient interview and review of hemodialysis charts for the following parameters: age, sex, smoking status, history of coronary artery disease (CAD), history of cerebrovascular disease (CVD), presence of peripheral vascular disease (PVD), history of diabetes mellitus (DM), history of fractures, etiology of renal failure, dialysis vintage, and adequacy of hemodialysis as measured by the most recently recorded single pool Kt/V calculated from the urea reduction ratio (URR). The presence of coronary artery disease was determined both by current symptoms (patient interview) using Canadian Cardiovascular Society functional classification of angina and history of cardiovascular events including acute coronary syndrome [21]. CVD included a history of cerebral vascular accident or transient ischemic attack. PVD was determined by the presence of claudication or peripheral revascularization surgery. Patients were considered current smokers if they had smoked at least one cigarette per day during the preceding six months, former smokers if they had abstained from smoking for at least one month after having smoked at least one cigarette per day for six months, and non-smokers if they had never smoked.

### Laboratory measures

Laboratory measures included ionized calcium (mmol/L) and phosphate (mmol/L) that were averaged over the three previous months, intact parathyroid hormone (PTH) (pmol/L), serum albumin (g/L), total cholesterol (mmol/L), LDL cholesterol (mmol/L), HDL cholesterol (mmol/L), and triglycerides (mmol/L). Intact PTH was assessed by means of electrochemiluminescence (Roche, Basel, Switzerland) modular analytics E170 immunoassay. Serum albumin was measured by the bromocresol green method (Roche, Basel, Switzerland). All of the aforementioned analyses were performed in the laboratory at Kingston General Hospital to minimize interlaboratory variability. Blood samples were stored at -80°C before the analysis of phylloquinone,%ucOC, and PIVKA-II. Fasting phylloquinone concentrations were measured using reversed-phase high-performance liquid chromatography [22, 23]. Osteocalcin (OC) was measured in serum by a radioimmunoassay that uses human OC for standard and tracer and a polyclonal antibody that recognizes intact OC and the large N-terminal midmolecule fragment. Uncarboxylated OC (ucOC) was determined in this assay as OC that does not bind *in vitro* to hydroxyapatite [23]. The ucOC is expressed as a percentage of the total OC that is not carboxylated (%ucOC), and a value >20% is consistent with subclinical vitamin K deficiency. This cutoff point is specific to this assay and should not be considered generalizable to other assays used for determination of %ucOC. PIVKA-II concentrations were determined by ELISA (Diagnostica Stago, Parsippany, NJ).

### Statistical analysis

All data were analyzed using the PASW software package (version 18.0, Chicago, IL). Descriptive statistics (mean ± SD for continuous data and frequency for categorical values) were generated for all variables. Spearman correlation coefficient was used to evaluate bivariate relationships among biomarkers of vitamin K status and continuous variables. To evaluate the relationship between vitamin K biomarkers and categorical variables, non-parametric tests including Mann Whitney U and Kruskal-Wallis H were applied as appropriate for two-level or multiple comparisons, respectively. Multivariable linear regression models were applied to determine independent predictors of vitamin K status using the backward-selection method. All statistical tests were two-sided, and an unadjusted *P* value of 0.05 or less was considered statistically significant.

## Results

Baseline demographic data for the study population are listed in Table 1. There were 44 HD patients with a mean age of 64.2 years (range 22 to 92 years) and mean dialysis duration of 43.8 ± 38.3 months (range 3 to 183 months) enrolled in this study.

**Table 1:** **Demographic and clinical features of study population**

Variables	N (%)	Mean *±* SD	Range
Clinical characteristics (n = 44)			
Age, years		64.2 *±* 14.5	22 to 92
Sex (male)	29 (65.9)		
Dialysis vintage, months		43.8 *±* 38.3	3 to 183
Etiology of ESKD			
Diabetes mellitus	11 (25.0)		
Renovascular disease	14 (31.8)		
Other	19 (43.2)		
Cardiovascular disease	14 (31.8)		
Peripheral vascular disease	17 (38.6)		
Cerebrovascular disease	6 (13.6)		
History of fracture	17 (38.6)		
Non-smoker	17 (38.6)		
Vitamin K status			
Phylloquinone concentration, nmol/L (n = 44)		1.25 *±* 1.17	0 to 5.3
%ucOC (n = 42)		24.5 *±* 15.4	5.2 to 63.1
PIVKA-II, nmol/L (n = 44)		3.98 *±* 2.51	1.5 to 14.5
Laboratory measures			
Phosphate, mmol/L (n = 43)		1.49 *±* 0.38	0.61 to 2.96
Calcium, ionized, mmol/L (n = 43)		1.17 *±* 0.08	0.97 to 1.36
Parathyroid hormone, pmol/L (n = 43)		38.7 *±* 39.4	0.9 to 220.1
Albumin, g/L (n = 43)		40.4 *±* 3.2	32 to 48
Total cholesterol, mmol/L (n = 43)		3.74 *±* 0.86	2.1 to 6.3
LDL cholesterol, mmol/L (n = 41)		1.62 *±* 0.63	0.4 to 3.1
HDL cholesterol, mmol/L (n = 43)		1.30 *±* 0.35	0.7 to 2.3
Triglycerides, mmol/L (n = 43)		1.75 *±* 1.23	0.8 to 5.6
Kt/V (n = 44)		1.52 *±* 0.35	0.71 to 2.23
URR (n = 44)		0.73 *±* 0.07	0.50 to 0.88

ESKD, End-stage kidney disease; %ucOC, percentage of undercarboxylated osteocalcin; PIVKA-II, Proteins induced by vitamin K absence or antagonism II; LDL, low-density lipoprotein; HDL, high-density lipoprotein; Kt/V, measure of dialysis adequacy, where *K* is dialyzer clearance of urea, *t* is dialysis time, and *V* is volume of distribution of urea; URR, urea reduction ratio.

The mean serum PIVKA-II level measured in 44 patients was 3.98 ± 2.51 nmol/L. Of these, 90.9% had subclinical vitamin K deficiency defined as PIVKA-II levels > 2 nmol/L. Higher PIVKA-II levels, corresponding to worse vitamin K status, were associated with increasing age, decreasing HDL and lower Kt/V, or inadequate dialysis (Table 2). There was no association between PIVKA-II levels and other lipid measures. Those with CAD had higher PIVKA-II levels on average (mean 5.16 ± 3.26 nmol/L) compared to those without CAD (mean 3.44 ± 1.90 nmol/L) (*P* = 0.009). There was a trend towards positive association with PVD and smoking status. The only independent predictor of PIVKA-II concentration was advancing age; for every 10-year increase in age, serum PIVKA-II levels increased by 0.5 nmol/L (Table 3). Those with CAD trended toward higher mean PIVKA-II levels in the multi-variable model, but this was not statistically significant.

**Table 2:** **Cross-sectional correlations among measures of vitamin K status, demographic and biochemical variables**

Variables		Phylloquinone		%ucOC		PIVKA-II	
		***r***	***p***	***r***	***p***	***r***	***p***
Demographic variables							
Age, years		0.108	0.49	0.063	0.69	0.37	0.015
Dialysis vintage, months		-0.25	0.11	0.06	0.72	-0.04	0.82
		***Mean ± SD***	***p***	***Mean ± SD***	***p***	***Mean ± SD***	***p***
Sex							
Male		1.27 *±* 1.23	0.91	25.4 *±* 16.6	0.87	3.93 *±* 2.47	0.98
Female		1.21 *±* 1.08		22.9 *±* 13.4		4.09 *±* 2.66	
Etiology of ESKD							
Diabetes mellitus		1.13 *±* 0.99	0.98	23.9 *±* 16.8	0.93	4.11 *±* 1.98	0.22
Renovascular disease		1.33 *±* 1.34		25.5 *±* 15.0		4.48 *±* 3.10	
Other		1.25 *±* 1.19		24.3 *±* 15.7		3.55 *±* 2.35	
Cardiovascular disease	Yes	1.15 + 1.01	0.72	19.8 *±* 13.7	0.12	5.16 *±* 3.26	0.009
	No	1.29 *±* 1.25		26.9 *±* 15.9		3.44 *±* 1.90	
Peripheral vascular disease	Yes	1.25 *±* 1.36	0.52	23.2 *±* 15.0	0.53	4.61 *±* 2.73	0.08
	No	1.24 *±* 1.06		25.4 *±* 15.9		3.59 *±* 2.33	
Cerebrovascular disease	Yes	1.15 *±* 1.15	0.84	16.0 *±* 14.2	0.048	5.20 *±* 3.93	0.58
	No	1.26 *±* 1.19		26.0 *±* 15.3		3.79 *±* 2.22	
History of fracture	Yes	0.94 *±* 0.85	0.20	19.6 *±* 14.8	0.035	4.08 *±* 2.87	0.55
	No	1.44 *±* 1.31		27.9 *±* 15.1		3.93 *±* 2.31	
Smoking status	Current	0.55 *±* 0.45	0.043	29.6 *±* 20.0	0.55	3.74 *±* 1.08	0.09
	Former	1.65 *±* 1.47		24.2 *±* 13.4		4.44 *±* 2.51	
	Never	1.12 *±* 0.84		22.4 *±* 15.4		3.59 *±* 2.98	
		***r***	***p***	***r***	***p***	***r***	***p***
Laboratory variables							
Phosphate, mmol/L		-0.05	0.77	0.30	0.06	0.15	0.35
Calcium, ionized, mmol/L		0.15	0.33	0.22	0.18	0.08	0.63
Calcium-phosphate product, mmol^2^/L^2^		0.06	0.69	0.40	0.009	0.20	0.19
PTH, pmol/L		0.05	0.76	-0.26	0.10	-0.05	0.74
Albumin, g/L		-0.003	0.99	-0.07	0.66	-0.07	0.65
Total cholesterol, mmol/L		0.43	0.004	0.14	0.39	0.14	0.37
LDL cholesterol, mmol/L		0.17	0.30	0.22	0.18	0.21	0.19
HDL cholesterol, mmol/L		-0.04	0.82	0.26	0.10	-0.31	0.046
Triglycerides, mmol/L		0.45	0.002	-0.13	0.42	0.19	0.22
Kt/V		0.11	0.48	0.15	0.35	-0.33	0.027
URR		0.04	0.80	0.05	0.73	-0.06	0.68

ESKD, End-stage kidney disease; PTH, Parathyroid hormone; LDL, Low-density lipoprotein; HDL, High-density lipoprotein; Kt/V, measure of dialysis adequacy; URR, urea reduction ratio.

**Table 3:** **Multivariable linear regression models for significant predictors of phylloquinone, %ucOC and PIVKA-II**

	*β*Coefficient	95% Confidence interval	*p*
Phylloquinone (R^2^ = 0.275, n = 42)			
Triglycerides	0.54	0.25 to 0.84	0.001
Total cholesterol	-0.036	-0.46 to 0.39	0.87
%ucOC (R^2^ = 0.14, n = 41)			
Phosphate	14.80	1.71 to 29.13	0.028
PTH	-0.14	-0.26 to -0.02	0.028
Coronary artery disease	-9.14	-18.73 to 0.46	0.061
PIVKA-II (R^2^ = 0.16, n = 43)			
Age, years	0.05	0.01 to 0.10	0.033
Coronary artery disease	1.40	-0.14 to 2.93	0.07

PTH, parathyroid hormone; %ucOC, percentage of uncarboyxlated osteocalcin; PIVKA-II, proteins induced by vitamin K absence or antagonism.

Overall mean serum phylloquinone concentration in 44 patients was 1.25 ± 1.17 nmol/L (normal range 0.4 to 2.4 nmol/L [24]). Six patients (13.6%) met criteria for subclinical vitamin K deficiency, with phylloquinone levels below the normal range. Higher phylloquinone concentrations were positively associated with serum triglyceride levels and total cholesterol levels but not with LDL or HDL levels (Table 2). Current smokers had lower levels of serum phylloquinone than former smokers or lifelong non-smokers. The single independent predictor of phylloquinone concentration by regression analysis was serum triglycerides; on average, for every 1.0 mmol/L increase in triglycerides, serum phylloquinone increases by 0.53 nmol/L (*P* = 0.001) (Table 3). There was no association between serum phylloquinone and age, CAD, or calcium and phosphate laboratory parameters.

Mean %ucOC in 42 patients was 24.5 ± 15.4%. Subclinical vitamin K deficiency criteria defined as >20% ucOC was met in 21 patients (51%). Higher %ucOC (and, therefore, poorer vitamin K status) was associated with a higher calcium-phosphate product (Table 2). Prior cerebrovascular events and a history of fractures were negatively associated with %ucOC. Higher levels of %ucOC were independently associated with increasing serum phosphate and lower serum PTH levels (Table 3). There was no association between %ucOC and age, sex, dialysis adequacy, or smoking status.

There was no association between any one biomarker of vitamin K status with the other measures of vitamin K status (Table 4). There were no significant associations between any of the biomarkers of vitamin K status and sex, etiology of ESKD, dialysis vintage, or serum albumin (Table 2).

**Table 4:** **Cross-sectional associations between biomarkers of vitamin K status**

Variables	Phylloquinone		%ucOC		PIVKA-II	
	***r***	***p***	***r***	***p***	***r***	***p***
Phylloquinone			0.09	0.58	-0.18	0.25
%ucOC	0.09	0.58			0.08	0.62
PIVKA-II	-0.18	0.25	0.08	0.62		

%ucOC, percentage undercarboxylated osteocalcin; PIVKA-II, Protein induced by vitamin K absence or antagonism II.

## Discussion

The present study confirms previous reports that a suboptimal vitamin K status is prevalent in patients with ESKD [10, 17, 25–27]. Based on ranges determined in healthy adults, over 90% of these HD patients had increased PIVKA-II levels, confirming that a high prevalence of subclinical vitamin K deficiency exists in this population, particularly in older patients. Although clinical outcome studies are lacking, PIVKA-II may be a good biomarker of vitamin K status in this particular population as its measurement is not influenced by kidney function [19], and it has been demonstrated to respond in a dose- and time-dependent manner to vitamin K2 supplementation in hemodialysis patients [20]. We found no associations between phylloquinone levels and the two estimates of functional vitamin K status in this study, which may reflect dysregulation in metabolism that is associated with uremia and directly impacts vitamin K biomarkers.

The results of this small study support those of a recent trial examining the effect of oral vitamin K_2_ supplementation on biomarkers of functional vitamin K status in a cohort of 53 hemodialysis patients [20]. In this trial, the mean PIVKA-II level at baseline prior to supplementation was 5.6 ng/mL (reference < 2 ng/mL), with 49 of 50 patients having levels above the cited reference range. Similarly, baseline levels of uncarboxylated osteocalcin and dephosphorylated-uncarboxylated MGP were 8.4-fold and 4.5-fold higher than population-matched controls, respectively. There was a significant dose- and time-dependent improvement in functional vitamin K indices over the 6-week supplementation period, with subsequent rebound in dephosphorylated-uncarboxylated MGP levels following cessation of therapy [20]. This study suggests hemodialysis patients respond to vitamin K_2_ supplementation in terms of favorable biomarker responses. However, trials and large longitudinal studies linking vitamin K supplementation to clinical outcomes are currently needed to clarify the role of the various biomarkers of vitamin K deficiency. Functional vitamin K deficiency as measured by levels of dephosphorylated-uncarboxylated MGP (dp-ucMGP) has been associated with increased all-cause and cardiovascular mortality risk in dialysis patients [10]. However, the dp-ucMGP fraction is responsive to vitamin K supplementation. The physiology and biochemistry of MGP will only be clarified by longitudinal analysis of vitamin K biomarkers in population studies.

In our study, the mean PIVKA-II concentration was 3.98 ± 2.51 nmol/L and, similar to the Westenfeld study [20], the overwhelming majority of patients (91%) met criteria for sub-clinical vitamin K deficiency. In our study, higher concentrations of PIVKA-II were associated with advancing age, the presence of cardiovascular and peripheral vascular disease and poorer dialysis adequacy; however in the multivariable model only age remained significant. By way of comparison, PIVKA-II levels were measured using identical techniques and in the same laboratory as this study in two previous reports. In a cohort of 416 community-dwelling elderly subjects, the mean (SD) of PIVKA-II at baseline was lower at 2.43 (0.06) nmol/L [28]. A second study assessed PIVKA-II concentrations in younger patients with rheumatoid arthritis prior to supplementation with vitamin E. The mean (SD) of PIVKA-II at baseline in this younger group was 1.7 (1.7) nmol/L [29].

Phylloquinone concentrations are believed to reflect tissue levels and are influenced by dietary intake. Based on ranges determined in healthy adults, we previously demonstrated in a larger cohort of HD patients that 29% had very low (<0.4 nM/L) circulating concentrations of phylloquinone [25]. In this smaller cohort, only 13.6% had very low levels, and the reasons for this difference are unknown but may reflect differences in vitamin K intake between the two cohorts, which were not captured. Similar to our previous study, there was a strong independent relationship between phylloquinone and triglyceride levels. Studies have shown that circulating phylloquinone is transported primarily by triacylglycerol-rich lipoproteins (including chylomicron remnants and very low density lipoproteins) following absorption, while little is carried by low-density or high-density lipoprotein fractions [30]. ESKD is associated with defects in reverse cholesterol transport and is typically characterized by elevated triglycerides and low HDL-cholesterol. Therefore, interpretation of circulating phylloquinone level as a biomarker of vitamin K status directly related to dietary intake in patients with ESKD is uncertain. However, in a cross-sectional study of prevalent HD patients, lower circulating levels of phylloquinone were the strongest predictor for the presence of a vertebral fracture, suggesting that phylloquinone level is relevant to bone health in this population [31].

Sixty percent of subjects had elevated concentrations of %ucOC and therefore met the criteria for sub-clinical vitamin K deficiency as it is defined in the general population. This is significantly greater than the mean %ucOC in the Framingham Offspring Study population (16.1% in males and 23.5% in post-menopausal women), in which the assessment of %ucOC was performed in the same laboratory. Total OC levels are significantly increased in patients with ESKD due to a combination of bone resorption and retention, but it is unknown if the γ-carboxylated fraction in circulation reflects vitamin K status in bone. Similar to our previous studies, we found strong association between %ucOC and parameters of mineral metabolism (*i.e.* elevated calcium-phosphorus product).

We found no associations between phylloquinone levels and the two estimates of functional vitamin K status in this study. In addition, our group and others have not demonstrated an association between phylloquinone and %ucOC in larger cohorts of patients with CKD [17, 25–27]. In contrast, longitudinal studies of vitamin K status performed in the general community-dwelling population generally report significant negative correlations between phylloquinone levels and both functional estimates of vitamin K status in liver (PIVKA-II) and bone (%ucOC). This discrepancy may reflect the cross-sectional nature of this small study, or alternatively it may highlight the limitations of using these biomarkers of vitamin K status in patients with ESKD in whom metabolic dysregulation can directly impact their estimates. In addition, the correlates of poor vitamin K status were different depending on the biomarker considered.

Vitamin K deficiency in the ESKD population likely relates, in part, to overall low caloric intake or dietary patterns that preclude the intake of vitamin K-rich foods. This was confirmed in a recent study of 40 hemodialysis patients that involved a 4-day food record [32]. The vitamin K1 was low in these patients (median 140 ug/day) compared to intakes reported in a reference population of healthy adults (median 200 ug/day). Although the daily Adequate Intake (AI) of vitamin K for adult men and women is 90 and 120 ug, respectively [33], this is the amount considered sufficient to prevent frank vitamin K deficiency and coagulopathy but may be inadequate for complete extra-hepatic VKDP carboxylation [12, 34]. In the ESKD population, a state of subclinical vitamin K deficiency resulting from prolonged nutritional deficiency may further accelerate their risk of VC and cardiovascular morbidity.

The small sample size and broad range in dialysis vintage observed in this cohort limit the conclusions that may be drawn. However, the results of this study support previous work that indicates there is a very high prevalence of subclinical vitamin K deficiency in the ESKD population. These patients therefore may be at risk for any biological consequences of vitamin K deficiency. There is an emerging role for vitamin K in oxidative stress reduction and a growing body of literature links poor vitamin K status with arterial calcification [35, 36]. One recent report in dialysis patients has linked the presence of dephosphorylated-uncarboxylated MGP in the circulation to calcification outcomes in patients with ESKD; however, no other biomarker of vitamin K status was simultaneously assessed in this study [36].

## Conclusions

In this study, we found that a significant proportion of HD patients met criteria for subclinical vitamin K deficiency. Of the biomarkers measured, PIVKA-II may be superior given its independence of renal function or dyslipidemia, both of which may confound the other vitamin K biomarkers. Studies in patients with ESKD linking biomarkers of vitamin K status to important patient outcomes, including cardiovascular disease, nutritional status and mortality, are required in order to determine the optimal biomarker for evaluating vitamin K status in this particular population.

## References

[1] Collins AJ, Li S, Ma JZ, Herzog C (2001). Cardiovascular disease in end-stage renal disease patients. Am J Kidney Dis.

[2] Tentori F (2010). Mineral and bone disorder and outcomes in hemodialysis patients: results from the DOPPS. Semin Dial.

[3] Raggi P, Bommer J, Chertow GM (2004). Valvular calcification in hemodialysis patients randomized to calcium-based phosphorus binders or sevelamer. J Heart Valve Dis.

[4] Chertow GM, Raggi P, McCarthy JT, Schulman G, Silberzweig J, Kuhlik A, Goodman WG, Boulay A, Burke SK, Toto RD (2003). The effects of sevelamer and calcium acetate on proxies of atherosclerotic and arteriosclerotic vascular disease in hemodialysis patients. Am J Nephrol.

[5] Block GA, Raggi P, Bellasi A, Kooienga L, Spiegel DM (2007). Mortality effect of coronary calcification and phosphate binder choice in incident hemodialysis patients. Kidney Int.

[6] Krasniak A, Drozdz M, Pasowicz M, Chmiel G, Michalek M, Szumilak D, Podolec P, Klimeczek P, Konieczynska M, Wicher-Muniak E, Tracz W, Khoa TN, Souberbielle JC, Drueke TB, Sulowicz W (2007). Factors involved in vascular calcification and atherosclerosis in maintenance haemodialysis patients. Nephrol Dial Transplant.

[7] London GM, Guerin AP, Marchais SJ, Metivier F, Pannier B, Adda H (2003). Arterial media calcification in end-stage renal disease: impact on all-cause and cardiovascular mortality. Nephrol Dial Transplant.

[8] Proudfoot D, Shanahan CM (2006). Molecular mechanisms mediating vascular calcification: role of matrix Gla protein. Nephrology.

[9] Krueger T, Westenfeld R, Ketteler M, Schurgers LJ, Floege J (2009). Vitamin K deficiency in CKD patients: a modifiable risk factor for vascular calcification?. Kidney Int.

[10] Schlieper G, Westenfeld R, Kruger T, Cranenburg EC, Magdeleyns EJ, Brandenburg VM, Djuric Z, Damjanovic T, Ketteler M, Vermeer C, Dimkovic N, Floege J, Schurgers LJ (2011). Circulating nonphosphorylated carboxylated matrix gla protein predicts survival in ESRD. J Am Soc Nephrol.

[11] Booth SL, Suttie JW (1998). Dietary intake and adequacy of vitamin K. J Nutr.

[12] Booth SL, Martini L, Peterson JW, Saltzman E, Dallal GE, Wood RJ (2003). Dietary phylloquinone depletion and repletion in older women. J Nutr.

[13] Booth SL, Lichtenstein AH, O’Brien-Morse M, McKeown NM, Wood RJ, Saltzman E, Gundberg CM (2001). Effects of a hydrogenated form of vitamin K on bone formation and resorption. Am J Clin Nutr.

[14] Booth SL, Broe KE, Peterson JW, Cheng DM, Dawson-Hughes B, Gundberg CM, Cupples LA, Wilson PW, Kiel DP (2004). Associations between vitamin K biochemical measures and bone mineral density in men and women. J Clin Endocrinol Metab.

[15] Szulc P, Chapuy MC, Meunier PJ, Delmas PD (1996). Serum undercarboxylated osteocalcin is a marker of the risk of hip fracture: a three year follow-up study. Bone.

[16] Szulc P, Arlot M, Chapuy MC, Dubeouf F, Meunier PJ, Delmas PD (1994). Serum undercarboxylated osteocalcin correlates with hip bone mineral density in elderly women. J Bone Miner Res.

[17] Kohlmeier M, Saupe J, Shearer MJ, Schaefer K, Asmus G (1997). Bone health of adult hemodialysis patients is related to vitamin K status. Kidney Int.

[18] Vergnaud P, Garnero P, Meunier PJ, Breart G, Kamihagi K, Delmas PD (1997). Undercarboxylated osteocalcin measured with a specific immunoassay predicts hip fracture in elderly women: the EPIDOS Study. J Clin Endocrinol Metab.

[19] Kato A, Yasuda H, Togawa A, Yamamoto T, Yonemura K, Maruyama T, Maruyama Y, Hishida A (2002). Measurement of des-gamma-carboxy prothrombin levels in hemodialysis patients positive for anti-hepatitis virus C antibody. Clin Nephrol.

[20] Westenfeld R, Krueger T, Schlieper G, Cranenburg EC, Magdeleyns EJ, Heidenreich S, Holzmann S, Vermeer C, Jahnen-Dechent W, Ketteler M, Floege J, Schurgers LJ (2012). Effect of vitamin K2 supplementation on functional vitamin K deficiency in hemodialysis patients: a randomized trial. Am J Kidney Dis.

[21] McGillion M, L’Allier PL, Arthur H, Watt-Watson J, Svorkdal N, Cosman T, Taenzer P, Nigam A, Malysh L (2009). Recommendations for advancing the care of Canadians living with refractory angina pectoris: a Canadian Cardiovascular Society position statement. Can J Cardiol.

[22] Davidson KW, Sadowski JA (1997). Determination of vitamin K compounds in plasma or serum by high-performance liquid chromatography using postcolumn chemical reduction and fluorimetric detection. Methods Enzymol.

[23] Gundberg CM, Nieman SD, Abrams S, Rosen H (1998). Vitamin K status and bone health: an analysis of methods for determination of undercarboxylated osteocalcin. J Clin Endocrinol Metab.

[24] Sokoll LJ, Sadowski JA (1996). Comparison of biochemical indexes for assessing vitamin K nutritional status in a healthy adult population. Am J Clin Nutr.

[25] Pilkey RM, Morton AR, Boffa MB, Noordhof C, Day AG, Su Y, Miller LM, Koschinsky ML, Booth SL (2007). Subclinical vitamin K deficiency in hemodialysis patients. Am J Kidney Dis.

[26] Holden RM, Iliescu E, Morton AR, Booth SL (2008). Vitamin K status of Canadian peritoneal dialysis patients. Perit Dial Int.

[27] Holden RM, Morton AR, Garland JS, Pavlov A, Day AG, Booth SL (2010). Vitamins K and D status in stages 3-5 chronic kidney disease. Clin J Am Soc Nephrol.

[28] Crosier MD, Peter I, Booth SL, Bennett G, Dawson-Hughes B, Ordovas JM (2009). Association of sequence variations in vitamin K epoxide reductase and gamma-glutamyl carboxylase genes with biochemical measures of vitamin K status. J Nutr Sci Vitaminol.

[29] Booth SL, Golly I, Sacheck JM, Roubenoff R, Dallal GE, Hamada K, Blumberg JB (2004). Effect of vitamin E supplementation on vitamin K status in adults with normal coagulation status. Am J Clin Nutr.

[30] Schurgers LJ, Vermeer C (2002). Differential lipoprotein transport pathways of K-vitamins in healthy subjects. Biochim Biophys Acta.

[31] Fusaro M, Noale M, Viola V, Galli F, Tripepi G, Vajente N, Plebani M, Zaninotto M, Guglielmi G, Miotto D, Dalle Carbonare L, D’Angelo A, Naso A, Grimaldi C, Miozzo D, Giannini S, Gallieni M, Vitamin K Italian (VIKI) Dialysis Study Investigators (2012). Vitamin K, vertebral fractures, vascular calcifications, and mortality: VItamin K Italian (VIKI) dialysis study. J Bone Miner Res.

[32] Cranenburg EC, Schurgers LJ, Uiterwijk HH, Beulens JW, Dalmeijer GW, Westerhuis R, Magdeleyns EJ, Herfs M, Vermeer C, Laverman GD (2012). Vitamin K intake and status are low in hemodialysis patients. Kidney Int.

[33] Institute of Medicine (2001). Dietary reference intakes for vitamin A, vitamin K1, arsenic, boron, chromium, copper, iodine, iron, manganese, molybdenum, nickel, silicon, vanadium and zinc.

[34] Binkley NC, Krueger DC, Kawahara TN, Engelke JA, Chappell RJ, Suttie JW (2002). A high phylloquinone intake is required to achieve maximal osteocalcin gamma-carboxylation. Am J Clin Nutr.

[35] Westhofen P, Watzka M, Marinova M, Hass M, Kirfel G, Muller J, Bevans CG, Muller CR, Oldenburg J (2011). Human vitamin K 2,3-epoxide reductase complex subunit 1-like 1 (VKORC1L1) mediates vitamin K-dependent intracellular antioxidant function. J Biol Chem.

[36] Schurgers LJ, Barreto DV, Barreto FC, Liabeuf S, Renard C, Magdeleyns EJ, Vermeer C, Choukroun G, Massy ZA (2010). The circulating inactive form of matrix gla protein is a surrogate marker for vascular calcification in chronic kidney disease: a preliminary report. Clin J Am Soc Nephrol.

